# Display of single-chain variable fragments on bacteriophage MS2 virus-like particles

**DOI:** 10.1186/s12951-016-0240-7

**Published:** 2017-02-13

**Authors:** Christopher A. Lino, Jerri C. Caldeira, David S. Peabody

**Affiliations:** Department of Molecular Genetics and Microbiology, University of New Mexico, Albuquerque, NM USA

**Keywords:** Virus-like particles, Phage display, Single-chain antibodies, Bacteriophage MS2

## Abstract

**Background:**

Virus-like particles (VLPs) of the RNA bacteriophage MS2 have many potential applications in biotechnology. MS2 VLPs provide a platform for peptide display and affinity selection (i.e. biopanning). They are also under investigation as vehicles for targeted drug delivery, using display of receptor-specific peptides or nucleic acid aptamers to direct their binding to specific cell-surface receptors. However, there are few molecules more suited to the precise targeting and binding of a cellular receptor than antibodies.

**Results:**

Here we describe a strategy for display of four different functional single-chain variable fragments (scFvs) on the surface of the MS2 VLP. Each scFv is validated both for its presence on the surface of the VLP and for its ability to bind its cognate antigen.

**Conclusions:**

This work demonstrates the suitability of the MS2 VLP platform to display genetically fused scFvs, allowing for many potential applications of these VLPs and paving the way for future work with libraries of scFvs displayed in a similar manner on the VLP surface. These libraries can then be biopanned and novel scFv binders to targets can be readily discovered.

## Background

Virus-like particles (VLPs) are finding diverse applications in nanobiotechnology, including as targeted drug delivery vehicles (see Ref. [1] for a recent review) and imaging agents (for some examples see Refs. [2–5]). Some applications, especially those requiring the VLP be directed to abnormal cells within a population of healthy cells, require the VLP to recognize receptors on specific cell types. Targeting can be mediated by decoration of the surface of the VLP with a peptide [6] or nucleic acid aptamer [7] specific for a receptor on the cell surface. Antibodies have also been utilized for this application [8–11] and can sometimes offer affinity and specificity advantages over other targeting moieties. Furthermore, a large number of antibodies with known specificities for diverse receptors have already been characterized, providing a rich source of potential targeting molecules. Single-chain variable fragments (scFvs) are modified versions of full antibodies that contain the antigen binding site, but they are also more amenable to genetic manipulation than the intact antibody because they are smaller and are comprised of a single polypeptide chain. Further, the absence of a fragment crystallizable (Fc) region helps prevent unwanted off-target binding to cells with Fc-receptors. Antibodies can be attached to a drug delivery vehicle (e.g. a VLP) by chemical conjugation [8–11], but display by genetic fusion to a VLP structural protein is an attractive alternative. Not only does genetic fusion offer production advantages, but at least in the case of the bacteriophage MS2 system described here (and previously described, see Refs. [12–14]), the VLP encapsidates the RNA that encodes it. The MS2 VLP platform has already been developed for display and affinity selection from random peptide libraries [12].

Here we describe the functional display of four genetically-fused scFvs on MS2 VLPs. The work presented here paves the way for display of random scFv libraries on the MS2 VLP, allowing for identification of scFvs with new binding affinities by biopanning directly on the VLP itself. Because of the simplicity of the MS2 VLP, the creation and biopanning of the scFv-MS2 library could be accomplished entirely in vitro, allowing for greater library complexities and faster selection/production than many other scFv display platforms.

## Methods

### Plasmids and proteins

Plasmids pDSP1, pDSP62 and their derivatives have been previously described [15]. The new constructs described here were made using standard molecular biology methods and have the characteristics described here.

**Fig. 1:**
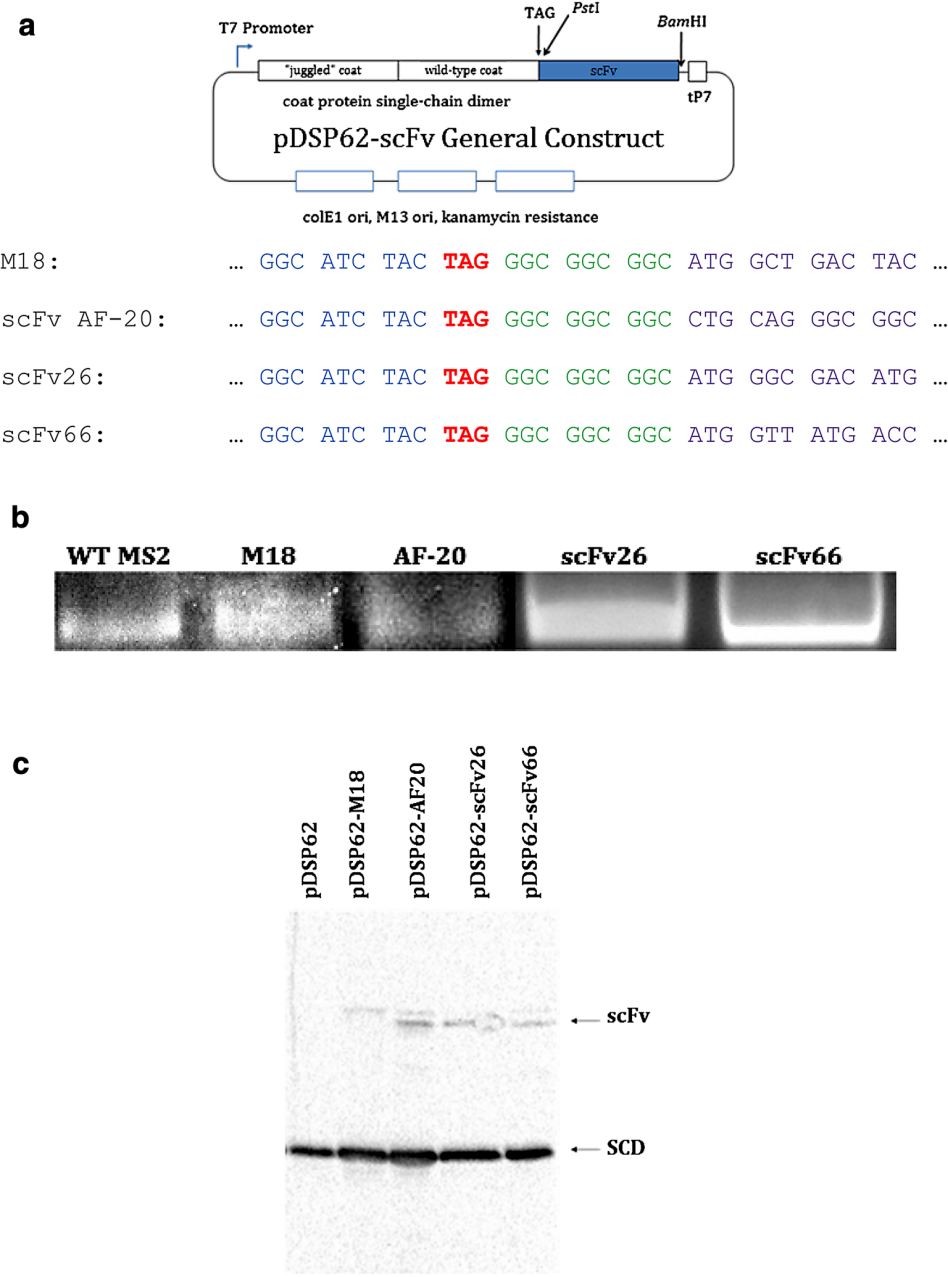
The architecture of plasmids used along with results of scFv-MS2 VLP fusion construct expression. **a** Schematic representation and abbreviated nucleotide sequence of pDSP62-scFv. In all cases, the specific scFv is fused in the same way to coat protein. Note the end of the MS2 coat protein sequence in *blue*, the amber stop codon in *red*, the flexible glycine linker between coat and scFv in *green*, and the beginning of the specific scFv sequence in *purple*. tP7 = T7 terminator (paired with leading T7 promoter). **b** Agarose gel stained with ethidium bromide indicating the presence of intact, RNA-containing VLPs in both wild-type (WT) and all fusion (M18, AF20, scFv26, scFv66) samples. **c** Western blot results for samples found in **b**. Proteins were separated via SDS-PAGE and then transferred to nitrocellulose for blotting. Blot was developed with rabbit anti-MS2 primary and goat anti-rabbit HRP-IgG secondary antibodies. Note the presence of the single-chain dimer (SCD) coat protein in all samples and a higher-weight band in the scFv fusion samples (scFv); this is presumed to be the scFv-coat protein fusion. Double bands in some wells are believed to be potential degradation products of the scFv-coat protein fusion, though their presence does not appear to affect function. pDSP62 = WT coat protein only

Each of the coat protein-scFv fusions was constructed using pDSP62AP, whose principle features are illustrated in Fig. 1a. The plasmid expresses coat protein in bacteria as a single-chain dimer [12, 13, 15–18] and confers resistance to kanamycin. The coat sequence is terminated with a single amber codon which is immediately followed by *Pst*I and *Bam*HI sites. Each of the scFvs utilized in these studies was assembled from synthetic oligonucleotides by Gibson assembly [19] and was flanked by *Bam*HI and *Pst*I sites for facile insertion into pDSP62AP. The scFv sequences were designed so that suppression of the stop codon results in the production of a coat protein single-chain dimer fused through an oligoglycine linker to the scFv. Low level stop codon suppression (a few percent) was promoted by an alanine-inserting amber-suppressing tRNA [20] expressed from the *lac* promoter on pNMsupA, a pACYC184 derivative [21]. A similar plasmid, pNMsupS2, expresses a serine-inserting amber-suppressing tRNA based on the *E. coli sup*D mutation [20, 22] and suppresses at high efficiency. We introduced an A → G mutation in the anticodon loop to reduce its suppression efficiency to a few percent. Since the suppressor-tRNA-producing plasmids represent a different incompatibility group, and because they confer resistance to a different antibiotic (chloramphenicol), they can be stably maintained in cells also containing pDSP62AP derivatives.

Proteins were produced by expression in *E. coli* strain C41(DE3) (Lucigen) and the resulting VLPs were purified by Sepharose CL4B column chromatography as previously described [13]. Purity of VLPs was assessed by electrophoresis on polyacrylamide gels (17.5%) in the presence of sodium dodecyl sulfate and by electrophoresis of the intact particle on 1% phosphate agarose gels. SDS gels were stained with Coomassie brilliant blue R250 and the proteins in duplicate gels were transferred to nitrocellulose membranes and probed with rabbit anti-MS2 serum and an alkaline phosphatase-conjugated goat anti-rabbit IgG secondary antibody. Agarose gels were stained with ethidium bromide to reveal the presence of the RNA-containing VLPs.

### ELISA

To determine whether the anthrax protective antigen-specific scFv M18 was functionally displayed on the VLP surface, it was assayed for its ability to mediate interaction of the VLP with protective antigen in ELISA. Wells of an Immulon 2 ELISA plate (Thermo Scientific) were coated with 500 ng of anthrax protective antigen (Invitrogen) by incubation at 4 °C overnight in PBS. Wells were blocked for 2 h at room temperature (RT) with 0.5% non-fat dry milk in PBS buffer, and then serial dilutions of either WT or M18-expressing VLPs were added to each well and incubated for 2 h at RT. Mouse anti-MS2 serum was added at a 1:2000 dilution to each well and incubated for 2 h at RT. After washing with PBS, the wells were incubated for 1 h at RT with horseradish peroxidase (HRP)-conjugated goat anti-mouse IgG at a 1:5000 dilution. After washing, the plates were developed with the chromogenic substrate 2,2′-azino-bis(3-ethylbenzthiazoline-6-sulfonic acid) (ABTS), and after sufficient color development, optical density at 405 nm was measured.

### Mammalian cell culture

Cell lines, media, and supplements were obtained from ATCC and cultivated according to the supplier’s instructions. Hep3B cells were maintained in culture plates in EMEM with 10% FBS. Thle-3 cells were grown in plates coated with BSA, fibronectin, and bovine collagen type I. The culture medium used was BEGM (gentamicin, amphotericin, and epinephrine were discarded from the BEGM Bullet kit) with 5 ng/mL epidermal growth factor, 70 ng/mL phosphatidylethanolamine, and 10% FBS. Vero cells (CCL-81) were maintained in DMEM with 10% FBS, and were passaged after treatment with 0.25% trypsin at a sub-cultivation ratio of 1:10. All cells were maintained at 37 °C in a humidified atmosphere (air supplemented with 6% CO_2_) and were passaged after treatment with 0.05% trypsin at a sub-cultivation ratio of 1:7.

### Fluorescence activated cell sorting (FACS)

VLPs (WT and AF-20) were labeled with Alexa Fluor 488 NHS ester (Invitrogen) on surface amines according to the manufacturer’s instructions. To reduce non-specific interactions with cells, VLPs were also derivatized at surface carboxyl groups using EDC (Pierce) and an aminated polyethylene glycol (PEG) 12mer. Cells (1 × 10^6^) were exposed to increasing amounts of VLPs (4 × 10^12^ to 4 × 10^15^, roughly 16 μg to 16 mg) for 1 h at 37 °C. Cells were then pelleted and washed in FACS buffer (1× PBS, 1% BSA, pH 7.4) before being fixed with 3.7% formaldehyde and resuspended in FACS buffer. They were immediately analyzed with a FACSCalibur flow cytometer (Becton–Dickinson) equipped with BD CellQuest software at the UNM Shared Flow Cytometry and High Throughput Screening Resource. Data were acquired with the FSC channel in linear mode and all other channels in log mode. Events were triggered based upon forward light scatter, and a gate was placed on the forward scatter-side scatter plot that excluded cellular debris. Samples were excited using the 488-nm laser source, and emission intensity was collected in the FL1 channel (530/30). Fluorescence intensity was determined using the BD CellQuest software and data were plotted using Graphpad Prism.

### Confocal microscopy

1 × 10^6^ cells/mL of either Hep3B or Thle-3 were seeded on sterile coverslips (25-mm, No. 1.5) coated with 0.01% poly-l-lysine and allowed to adhere for 4–24 h at 37 °C. 10 μg (~2.4 × 10^12^ particles) of either WT or AF-20 VLPs were incubated with the cells for 2 h at 37 °C, washed three times with 1× PBS, fixed with 3.7% formaldehyde (10 min at RT), and mounted with SlowFade Gold. Prior to fixation, cells were stained with CellTracker Red CMFDA (Invitrogen) to visualize cytoplasm and Hoechst 33342 (Invitrogen) to visualize the nucleus. Three-color images were acquired using a Zeiss LSM510 META (Carl Zeiss MicroImaging, Inc.) operated in Channel mode of the LSM510 software; a 63×, 1.4-NA oil immersion objective was employed in all imaging. Typical laser power settings were: 30% transmission for the 405-nm diode laser, 5% transmission (60% output) for the 488-nm Argon laser, 100% transmission for the 543-nm HeNe laser, and 85% transmission for the 633-nm HeNe laser. Gain and offset were adjusted for each channel to avoid saturation and were typically maintained at 500–700 and −0.1, respectively. 8-bit z-stacks with 1024 × 1024 resolutions were acquired with a 0.7 to 0.9-μm optical slice. LSM510 software was used to overlay channels and to create 3D projections of z-stack images.

### Neutralization of Nipah-VSV pseudotype

Neutralization assays were performed using the method of Tamin et al. [23]. Briefly, Vero cells were grown in culture flasks to approximately 80% confluence and passaged after treatment with 0.25% trypsin. Cells were seeded in DMEM with 10% FBS onto pre-treated 96-well plates at a concentration of 8000 cells/well and allowed to adhere for approximately 18 h at 37 °C. VLP samples starting at a concentration of 1 µg/µl (1.3 × 10^12^ total particles) were diluted fourfold serially, and each was incubated for 30 min at room temperature with a VSV recombinant virus pseudotyped with NiV G- and F-protein and expressing *Renilla* luciferase (NiVpp). The pseudotype-serum mixture was then added to the adhered Vero cells in the 96 well plate, and the cells were incubated with the mixture for 90 min at 37 °C. Cells were then washed three times with 1× D-PBS and 150 µl of DMEM with 10% FBS were added. After incubation for 18 h at 37 °C, cells were washed three times with 1× D-PBS, and then lysed and assayed for luciferase activity using the *Renilla* Luciferase Assay System (E2810) (Promega). Luminosity was read with a luminometer integrated over 10 s with a 2 s delay.

## Results

We chose four different scFvs to test the display capabilities of MS2 VLPs. One, called M18, recognizes the protective antigen (PA) of anthrax [24] and is based the nucleotide sequence given by Young and Collier [25]. Another, called scFv AF-20, is specific for a protein (AF-20) found abundantly on hepatocellular carcinoma cells (HCC) but not on hepatocytes [10]. We synthesized the anti-AF-20 scFv (henceforth referred to only as AF-20 for clarity within the text) from the nucleotide sequence given by Yeung [26]. Two other scFvs were derived from antibodies specific for proteins of Nipah virus. One (scFv26) recognizes the viral envelope (or G) protein [27], and the other (scFv66) binds the viral fusion (or F) protein [28]. Both anti-Nipah scFvs were based on previously described monoclonal antibodies [29]. All scFv coding sequences were synthesized by assembly PCR from synthetic oligonucleotides [19]. Each was flanked by unique *Pst*I and *Bam*HI sites for facile insertion into pDSP62AP (Fig. 1) and fusion to the coat protein C-terminus. We utilized the single-chain dimer version of coat protein [12, 16–18] because its higher thermodynamic stability makes it more tolerant of a variety of mutational perturbations, including peptide fusions and insertions.

From the outset, we assumed that high-level synthesis of the coat-scFv fusion protein would be problematic. Not only are properly-folded scFvs notoriously difficult to express in *E. coli* [30], but the presence of an scFv on every copy of single-chain dimer coat protein (90 scFvs total) is likely to interfere with VLP assembly by steric crowding at the capsid’s threefold symmetry axes. To limit the number of scFvs per VLP, we terminated the coat sequence with a single amber codon. Normally, ribosomes would efficiently terminate to produce the coat protein single-chain dimer. However, in the presence of an appropriate suppressor tRNA expressed from a second plasmid (i.e. pNMsupA or pNMsupS), nonsense suppression allows production of the fusion protein. The ratio of coat protein to coat-scFv fusion is a function of the efficiency of the nonsense suppressor. When the suppression efficiency is low, the coat protein itself is produced at high levels, but only a small percentage is fused to an scFv. The excess of single-chain coat protein thus produced co-assembles with the scFv fusion protein to produce a VLP that displays the antibody fragments at low copy number. Indeed, when the stop codon between coat protein and the scFv was removed, or suppressed by a highly efficient suppressor, a high level of the coat protein-scFv fusion was synthesized, but no VLPs were produced (not shown). Moreover, the majority of the fusion protein was found in the insoluble fraction of cell lysates, indicating a failure to properly fold. For the experiments described below, we used an alanine-inserting suppressor tRNA described by Miller [31]. Based on the relative amounts of fusion and non-fusion proteins produced, we estimate its suppression efficiency in our system to be about 3%, corresponding to an average of three copies of coat protein-scFv fusion per assembled VLP.

### Expression of scFv-MS2 fusion VLPs

The coat protein-scFv fusions were expressed in *E. coli* and purified by size-exclusion chromatography as described previously [13]. A schematic representation of the construct used for expression and purification, along with some relevant features of individual constructs, are shown in Fig. 1a. Each recombinant produced a coat protein whose elution behavior from Sepharose CL-4B was consistent with a particle the size of the MS2 VLP (not shown). Agarose gel electrophoresis verified the production of a particle with electrophoretic mobility very similar to that of the normal VLP. The fact that the band seen on the gel was susceptible to staining with ethidium bromide indicates the presence of an intact, closed VLP able to contain RNA (Fig. 1b). The proteins were subjected to electrophoresis in polyacrylamide gels containing SDS and then blotted to a nitrocellulose membrane. Probing with anti-MS2 serum shows that most of the protein takes the form of the single-chain dimer, and that smaller amounts of the fusion proteins are produced (Fig. 1c). The presence of the larger species depends on the scFv fusion construct and is therefore most likely to represent the coat protein-scFv fusion. This is due to the molecular weight of the scFv, which dramatically increases the molecular weight of the single-chain dimer.

### Functional testing of M18 VLPs

M18 is an scFv specific for the protective antigen (PA) of *Bacillus anthracis*, a widely-studied pathogen due to both the ease with which it can be produced and its potential applications as a bioweapon. To show that the scFv was displayed on the VLP in a functional form, we measured the ability of varying amounts of either wild-type (WT) or M18 VLPs to bind protective antigen adsorbed to the wells of an ELISA plate. The presence of the VLP was detected by reaction with rabbit anti-MS2 antibody, followed by goat anti-rabbit IgG conjugated to HRP. After reaction with 2,2′-azino-bis(3-ethylbenzthiazoline-6-sulfonic acid) (ABTS), the wells were read at an optical density of 405 nm on a plate reader. The results are shown in Table 1. In spite of the relatively high backgrounds with control VLPs, it is clear that the M18-VLP binds the antigen.

**Table 1:** ELISA results for WT and M18 VLPs against anthrax protective antigen

	OD_405_
Volume M18 VLP (μl @ 5 mg/ml)	
10	1.265
5	0.767
2.5	0.555
1	0.304
Volume WT VLP (μl @ 5 mg/ml)	
10	0.267
5	0.232

Varying amounts of WT VLPs (50–25 µg, 1.2 × 10^13^ to 6 × 10^12^ particles) and M18 VLPs (from 50–5 μg, 1.2 × 10^13^ to 1.2 × 10^12^ particles) were incubated on wells to which 500 ng of anthrax protective antigen (APA) was adsorbed. Wells were then probed with rabbit anti-MS2 primary and goat anti-rabbit HRP-IgG secondary antibodies, and the developing reagent used was ABTS. Numbers given in the table are optical densities (ODs) at 405 nm.

### Functional testing of AF-20 VLPs

**Fig. 2:**
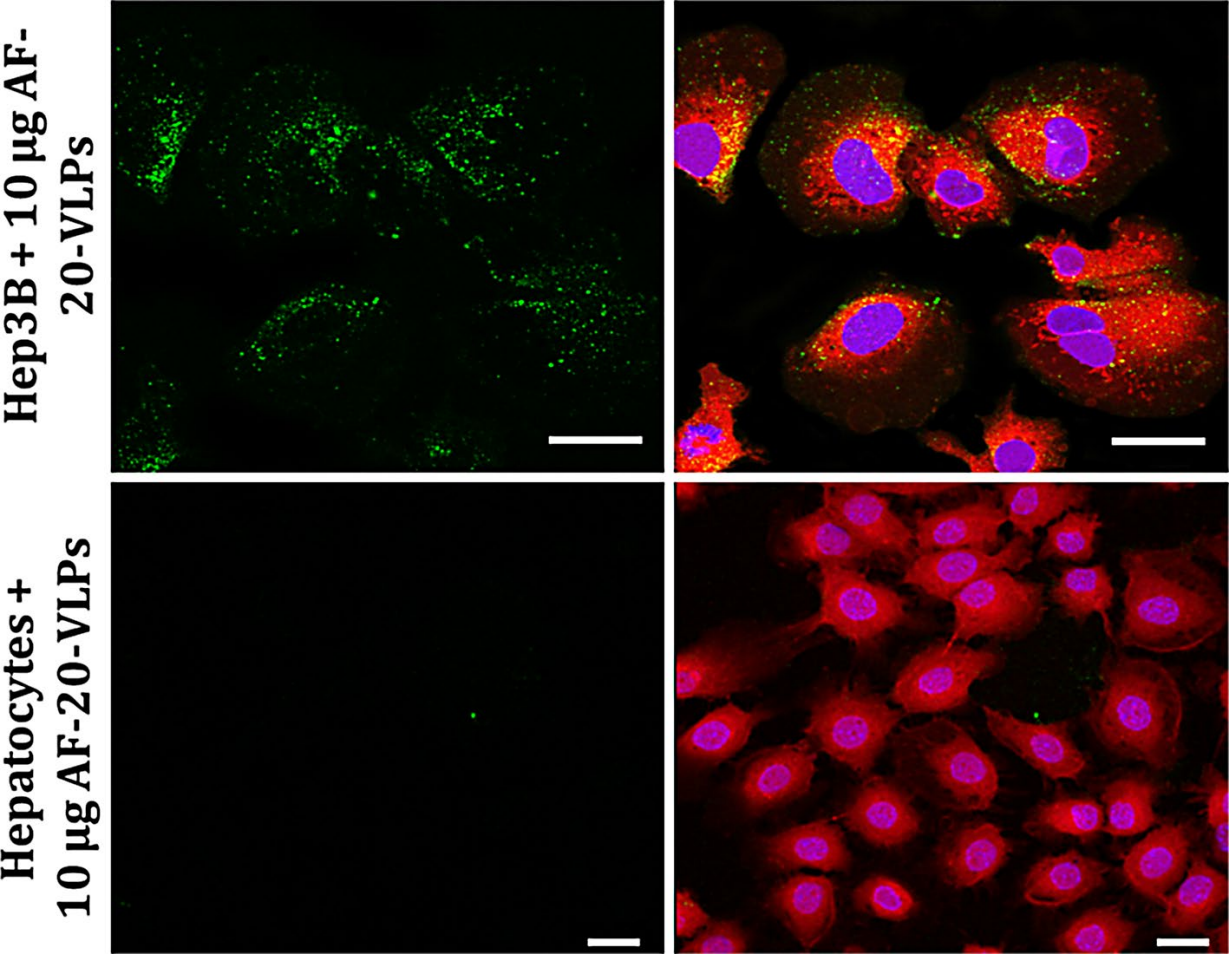
Confocal microscopy of AF-20-bearing VLPs to Hep3B and Thle-3 cells. Cells at 70% confluency were incubated with 10 µg (2.4 × 10^12^ particles) of AlexaFluor 488-labeled AF-20-bearing VLPs for 2 h at 37 °C in serum-free EMEM. Cells were then fixed with 3.7% formaldehyde and stained with CellTracker Red CMFDA to visualize the cytoplasm and Hoechst 33342 to visualize the genetic material in the nucleus. Cells were then imaged with a Zeiss LSM510 META confocal microscope. Note the abundance of AF-20-bearing VLPs bound to Hep3B but not Thle-3 cells. Examination of z-stacks of images also indicated some internalization of bound particles in the Hep3B sample (not shown). *Scale bars* 10 μm

As a second example of scFv display on VLPs, we chose an antibody specific for the AF-20 antigen found on a wide variety of HCC. Since no soluble form of AF-20 antigen is presently available [10], determining binding by ELISA was not possible. Instead, we labeled both WT and AF-20 VLPs with AlexaFluor 488 and compared their binding to Hep3B cells, which express AF-20 on their surfaces, with Thle-3 cells, which do not (Fig. 2). VLPs displaying the anti-AF-20 scFv abundantly bind Hep3B but not Thle-3 cells. In addition, Z-stack analysis shows that some of the VLPs were internalized by Hep3B cells, which is expected when AF-20 antigen interacts with AF-20 antibody [10] (not shown).

**Fig. 3:**
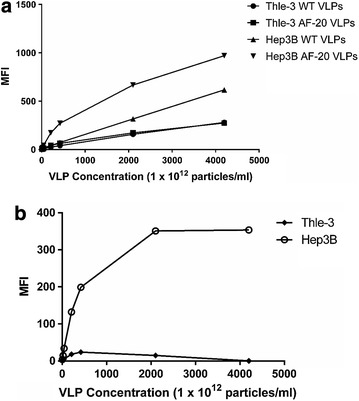
Binding of WT and AF-20 VLPs to Thle-3 and Hep3B cells vis FACS. The key of the figure indicates which VLP and cell type is being analyzed. **a** 1 × 10^6^ of either Thle-3 or Hep3B cells were incubated with increasing quantities of either WT or AF-20 AlexaFluor 488-labeled VLPs (4 × 10^12^ to 4 × 10^15^, roughly 16 µg to 16 mg) for 1 h at 37 °C. Cells were then fixed and washed, and mean fluorescent intensity (MFI) was measure via FACSCalibur. Note that neither WT nor AF-20 VLPs bind particularly well to Thle-3 cells (negative for AF-20 antigen), whereas only AF-20 VLPs bind to Hep3B cells (positive for AF-20 antigen) until the number of particles overwhelms the system and creates non-specific binding effects. **b** For each cell type from **a**, the value obtained for WT VLPs was treated as “background” binding and was subtracted from the value obtained for AF-20 VLPs, which were considered “signal”. This aides in the visualization of increased specific binding of AF-20 VLPs to Hep3B cells before total particle number becomes too large and the signal plateaus

We confirmed binding to Hep3B cells by flow cytometry. 1 × 10^6^ Thle-3 or Hep3B cells were exposed to increasing amounts of either WT or AF-20 VLPs (4 × 10^12^ to 4 × 10^15^ total particles) labeled with Alexa Fluor 488 for 1 h at 37 °C. Cells were then pelleted, washed, fixed with 3.7% formaldehyde, and then resuspended in FACS buffer. Cell samples were immediately analyzed with a FACSCalibur flow cytometer. The mean fluorescence intensity (MFI) was plotted for each of the four combinations of VLPs and cell types (Fig. 3a). Both WT and AF-20 VLPs showed a lower binding affinity to Thle-3 cells. However, Hep3B cells bind the scFv-displaying particles better than they bind WT VLPs, especially at low concentrations before non-specific interactions become important due to extremely high ratios of particles to cells. In Fig. 3b, background binding is determined for each cell type by removing the WT VLP signal from the AF-20 VLP signal. From this graph, we can visually confirm that WT VLPs do not bind any better to Hep3B cells than to Thle-3 cells. However, AF-20 VLPs demonstrate much greater binding specificity, plateauing finally at very high particle numbers.

### Functional testing of scFv26 and scFv66 VLPs

Nipah virus (NiV) is a BSL-4 agent that typically causes fatal respiratory and encephalitic infections in humans. Antibody neutralization of NiV can be conveniently determined, however, using a BSL-2 recombinant vesicular stomatitis virus (VSV) pseudotyped with NiV structural proteins. scFv26 is specific for Nipah G protein, a critical protein for viral attachment to host cells [27]. scFv66 is specific for the Nipah F protein, which mediates fusion to host cells [28]. Both of these scFvs neutralize NiV by binding to and inhibiting the function of their cognate proteins on the surface of the virus. VLPs displaying either scFv26 or scFv66 were tested for their ability to neutralize infection of Vero cells by the aforementioned pseudotyped VSV (known as NiVpp in the text) that had been engineered to express luciferase upon successful infection of cells.

**Fig. 4:**
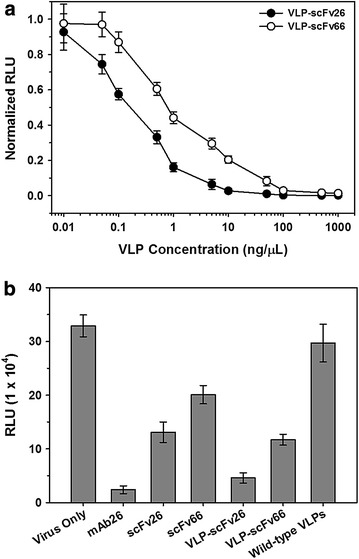
Neutralization of Nipah-pseudotyped VSV (NiVpp). **a** Varying concentrations of VLPs (0.1 μg/ml to 1 mg/ml, 1.3 × 10^8^ to 1.3 × 10^12^ total particles/well) were incubated with enough NiVpp (designed to express Nipah G- and F-proteins and express *Renilla* luciferase) to produce 300,000 relative light units (RLU) in control wells in this assay. This mixture was then used in an infection of Vero cells, and RLU due to infection of cells by non-neutralized VSV (and are thus producing luciferase) are measured in each case. There is concentration-dependent neutralization in both cases, though neutralization by scFv26 VLPs is slightly better than neutralization by scFv66 VLPs. **b** A constant 1.5 ng/μl concentration (corresponding to ~2.0 × 10^10^ particles for the scFv-VLP fusions) of each potential neutralizer of NiVpp (shown on the horizontal axis) was incubated with NiVpp and used in an infection of Vero cells as in **a**. RLU was again measured from luciferase expression to determine neutralization. *mAb26* G-specific monoclonal antibody (scFv parent), *scFv26* NiVG-specific scFv, *scFv66* NiVF-specific scFv, *VLP-scFv26/scFv66* the two scFvs fused to MS2 VLPs

Varying amounts of scFv26 or scFv66 VLPs were incubated with a constant amount of virus, which was then used to infect Vero cells. The luciferase produced corresponds to infection with the pseudotyped VSV, as only cells that have been infected will produce luciferase. Figure 4 shows that both particles efficiently inhibit (though do not entirely eliminate) infection, although scFv26 VLPs are slightly better (Fig. 4a). In fact, the VLP-associated scFv26 is nearly as effective as mAb26 itself and is far better than soluble scFv26 (Fig. 4b). Soluble scFv66 is an even weaker neutralizer than soluble scFv26, but is more effective when displayed on the VLP surface (Fig. 4b). The mAbs themselves seem to depend on their bivalency for efficient neutralization. The linkage of our estimated average of three copies of the scFvs to each VLP apparently restores the valency needed for potent neutralizing activity.

## Discussion

In this study, we constructed four scFvs: M18, AF-20, scFv26, and scFv66. In all four cases, the scFv was fused to the coat protein of MS2 via genetic insertion and displayed on the surface of the MS2 VLP. In each case, we showed that the scFv was both present and functional on the surface of the VLP. We limited the number of scFvs on the VLP surface via a system of nonsense suppression. By separating the coat protein and the C-terminally-fused scFv with an amber stop codon, we can control the number of coat-scFv fusion proteins that are created. We determined that roughly three copies of any given scFv are present on the surface of the VLP using this display method in our system.

We found it necessary to limit the number of scFvs displayed on the VLP surface because, when the scFv-MS2 fusion is expressed at high levels, the hybrid protein fails to fold properly. This means that populations consisting entirely of fusion protein don’t make VLPs, and all of the fusion protein ends up in the insoluble fraction of cell lysates. For most applications, we suspect that the relatively low valency of scFv display is a desirable characteristic, since high valency may show increased avidity for normally low-affinity targets, thus increasing non-specific binding. However, it may be desirable in some instances to present the scFv at high copy numbers. In such cases, it would be necessary to utilize better-folding scFv variants, or *E. coli* strains that are better adapted to the folding of heterologous proteins. For example, the cytoplasm of *E. coli* is normally reducing, meaning that the disulfide bonds necessary for correct folding and stability of antibodies are unable to form. Mutant strains that produce an oxidizing environment seem to facilitate antibody folding and might also enhance the folding of the coat-scFv fusion protein [32]. Strains that overproduce specific chaperone proteins might also be useful [33]. It would also be possible to display additional copies of the scFv on the VLP surface by means of chemical, rather than genetic, linkage. This destroys the phenotype-to-genotype link that genetic insertion affords, but once the sequence of a particular scFv is known, it can be synthesized in large quantities outside of the system and then attached to the surface of the VLP. Alternatively, it may be desirable to utilize a different coat protein as the basis of the VLP. For example, the coat protein of the *Acinetobacter* phage AP205 seems to be more tolerant of fusions at its termini. It has already been used for display of a few larger peptides and even of some small proteins, including an affibody [34, 35].

The ability to engineer the display of diverse scFvs on VLPs suggests a number of potential applications, including the targeted delivery of drugs and imaging agents. Nanoparticles possessing certain binding specificities should also find applications in diagnostics, as molecular detectors, and as potential treatments for disease. For example, as we showed here, VLPs displaying anti-NiV scFvs can potentially neutralize authentic NiV because they are capable of neutralizing NiV-pseudotyped VSV (NiVpp) with potencies near those of the corresponding monoclonal antibodies themselves. Presumably, this is due to the avidity effects associated with simultaneous display of several copies of the scFv on each VLP. In addition, the larger size of the VLP (when compared to the Fc portion of an antibody) may help two (or more) antibody binding sites gain access to adjacent proteins on the virus surface. Importantly, there may be additional room for improvement with these results, such as optimization of scFv copy number on the VLP surface. Such optimizations could lead to the scFv-VLP particles becoming even more potent neutralizers of NiV.

Additionally, as we observed in with the AF-20 VLPs, the scFv-MS2 fusion can be labeled with a dye (e.g. an Alexa Fluor NHS ester) without disrupting the function of the scFv. In this way, the VLPs displaying anti-NiV scFvs could also be used as detection reagents for NiV-infected cells, which display Nipah proteins on their surfaces as the virus buds. Finally, using the MS2 VLP has several advantages as a targeted drug delivery vehicle. It has a large interior volume, which can be loaded with a variety of molecular cargos. Indeed, several groups have already demonstrated the ability of MS2 VLPs to accept a variety of molecular cargos and then deliver them to specific cell types when decorated with appropriate targeting peptides or RNA aptamers [6–8]. The ability to engineer the display of a variety of different scFvs should allow the use of the huge collection of cell-specific antibodies now available to serve as the basis for generation of imaging and drug delivery vehicles for diverse cell types. Depending on the receptor targeted by the scFv, a wide variety of cell types and cell entry pathways could be targeted.

Another natural use for the display of scFvs on the surface of MS2 VLPs is the creation of scFv libraries that can be used in affinity selection experiments to discover novel scFv binders to a specific target. There are many systems already in the literature for the display of scFvs, including mammalian cells [36] and yeast [37]; several groups have even created scFv libraries for display on filamentous phage [38]. However, the MS2 system has several advantages over these systems. The first is its simplicity, owing to the nature of the VLP itself. The VLP is comprised of a single protein that can be expressed off of a plasmid, and this expression need not happen within a living cell. This means that libraries of scFvs displayed on the surface of MS2 VLPs could theoretically be transcribed and translated entirely in vitro, which would allow for even greater library complexities and speed/scale of production. In addition, owing to the properties of the VLP discussed above, the scFv-VLP fusion could be used directly after affinity selection for e.g. imaging or potential targeted delivery; there is no need to move to a different expression platform.

## Conclusions

Overall, we believe that the work presented here has direct implications in the field of nanobiotechnology. We have here displayed four different functional scFvs on the surface of the MS2 VLP, thus providing evidence of the suitability of the MS2 VLP platform to display genetically-fused scFvs. This allows for many uses of the scFv-MS2 VLP, including as detection reagents, imaging agents, and therapeutics. This work also paves the way for future work with libraries of scFvs displayed on the MS2 surface, allowing for biopanning and the discovery of novel scFvs to a target that can be utilized immediately within the platform that they were discovered.
